# Ability of an intentionally smaller anterior than posterior gap to reduce the sagittal tibial slope in opening wedge high tibial osteotomy

**DOI:** 10.1186/s12891-016-1066-z

**Published:** 2016-05-18

**Authors:** Seung-Beom Han, Hyung-Jun Park, Dae-Hee Lee

**Affiliations:** Department of Orthopaedic Surgery, Korea University, College of medicine, Anam hospital, Seoul, Korea; Department of Orthopaedic Surgery, Samsung Medical Center, Sungkyunkwan University School of Medicine, 81 Irwon-ro, Gangnam-gu, Seoul, 135-710 Korea (ROK)

**Keywords:** High tibial osteotomy, Navigation, Opening gap, Posterior slope, 3D-CT

## Abstract

**Background:**

We utilized in vivo 3- dimensional (D) computed tomography (CT) to determine whether the preoperatively planned anterior and posterior opening gap heights correlated with the real gaps following opening wedge high tibial osteotomy (HTO), as well as the relationships between anterior and posterior gap heights and change in sagittal tibial slope.

**Methods:**

This prospective study involved 41 patients (41 knees) undergoing navigation HTO for primary medial osteoarthritis. Mechanical axis (MA), weight-bearing line (WBL) ratio, and posterior tibial slope were measured on radiographs preoperatively and after 3 months. The anterior and posterior opening gaps created by osteotomy were measured using in vivo 3D CT and the patients were classified into a larger anterior or posterior gap group.

**Results:**

Of the 41 patients, 24 (59 %) had larger anterior and 17 (41 %) had larger posterior gaps. There were no between group differences in preoperative and postoperative slopes, or in change in slope. The correlation between preoperatively planned and postoperative posterior gaps was good, whereas the correlation of anterior gaps was only fair. Bland-Altman plots showed poor agreement for both preoperative and postoperative anterior and posterior gaps. The mean systematic difference (bias) was 2.3 mm (*p* < 0.001) for anterior and -1.0 mm (*p* = 0.033) for posterior gaps.

**Conclusions:**

Preoperatively calculated opening gaps, which were planned to be larger posteriorly than anteriorly to minimize the change in slope after surgery, did not correspond with postoperative opening gaps on 3D CT. In addition, postoperative tibial slope did not increase, even when the anterior gap was larger than the posterior gap.

**Trial registration:**

Trial registration number: KCT0001905, April 29, 2016.

## Background

High tibial osteotomy (HTO) is the established treatment for medial osteoarthritis with varus deformity [1, 2]. Open-wedge HTO is preferred to closed wedge HTO [3, 4] because closed wedge HTO gives rise to complications, including peroneal palsy and injury to the proximal tibiofibular joint and lateral ligaments [5, 6], whereas open-wedge HTO is easier to perform and more adjustable [7–9]. Despite the ability of open wedge HTO to correct varus deformities on the coronal plane, medial opening wedge HTO can inadvertently increase tibial slope in the sagittal plane [3, 10–12]. Increased tibial slope after open wedge HTO would increase the anterior translation of the tibia and increase tension in the anterior cruciate ligament. This unintended increase in tibial slope was found to be due to the unique anatomical configuration of the proximal tibial cross-section, such that an opening wedge with equal anterior and posterior gap heights can increase the sagittal tibial slope [13, 14]. Therefore, to dampen any increase in tibial slope, it has been recommended that the anterior opening gap should be about 1/2 to 2/3 of the posterior opening gap [13–15]. However, it is unclear whether all knees that undergo opening wedge HTO have smaller anterior than posterior gap heights. In addition, the theoretical correlations between anterior and posterior opening gap heights and changes in tibial slope after opening wedge HTO have not been verified in vivo. We therefore investigated the correlations between preoperatively planned anterior and posterior opening gap heights and actual gaps following opening wedge HTO, and we evaluated the relationships between anterior and posterior gap heights and changes in sagittal tibial slope, using in vivo three-dimensional computed tomography (3D-CT). We hypothesized that, during navigation-assisted open wedge HTO, preoperatively planned anterior and posterior gap heights correlate with postoperative 3D CT results, and that a larger anterior than posterior gap after surgery may result in a postoperative posterior tibial slope larger than the preoperative tibial slope.

## Methods

All patients with primary medial osteoarthritis who were candidates for navigation-assisted open wedge HTO at our institution between 2010 and 2011 were evaluated. Patients included were those who underwent navigation assisted open wedge HTO for medial osteoarthritis with varus deformity, as well as allografting and plate fixation. Patients considered ineligible for HTO included those aged >65 years and those with genu valgum, symptomatic osteoarthritis of the patellofemoral joint and lateral compartment, rheumatoid arthritis, knee range of movement <100°, lateral collateral ligament laxity of grade 3 or higher, and a flexion contracture >10°. Of the 45 patients (45 knees) initially approached, all agreed to take part in the study. After assessing eligibility, 43 patients (43 knees) were enrolled. After excluding two patients who did not undergo immediate postoperative 3D-CT, we evaluated 41 patients. The study protocol was approved by Korea University Anam Hospital Institutional Review Board (AN11132), and all patients provided written informed consent.

### Surgical techniques

Osteotomy and intraoperative analysis of the mechanical axis (MA) was performed using the Orthopilot® HTO 1.4 (B. Braun Aesculap, Tuttlingen, Germany) navigation system. All surgery was performed by a single surgeon. The transmitting arrays were fixed on the distal femur and the distal tibia using bicortical screws and another reference marker was wrapped around the foot with a rubber strap. The centers of the hip, knee, and ankle joints were marked, and the range of motion of each joint was assessed using a kinematic navigation system. Anatomical landmarks, such as the center of the patella, the medial and lateral epicondyles, the medial and lateral malleolus, the central point of the ankle and the medial point of the tibial plateau, were registered transcutaneously with a pointer. The computer can then form a model of the limb and calculate the MA. This procedure provides a preoperative assessment of varus deformity in degrees and the point of intersection of the MA on the tibial plateau. The mechanical leg axis was continuously monitored visually throughout the procedure. Each osteotomy was planned to commence at the medial tibial cortex along the metaphyseal flare (approximately 3-4 cm distal to the joint line) and was angled such that it terminated at the level of the tip of the fibular head laterally. The osteotomy was propagated through the proximal aspect of the insertion of the patellar ligament, leaving most of the ligament attached to the distal tibial fragment. Under the visual aid of an image intensifier, a guidewire was inserted in the above-described plane and the osteotomy was performed using a saw, for a distance of up to 1 cm from the lateral cortex. Thin osteotomes were used to complete the osteotomy just short of the lateral cortex, keeping the lateral cortex and lateral capsular hinge intact. The bone at the site of the osteotomy was forced open gradually while continuously assessing the MA by navigation. The navigation module measured the intersection of the MA with the tibial plateau, as well as the mechanical femorotibial axis following osteotomy. When the MA passed through 62 % of the tibial plateau, the osteotomy was stabilized using a fixed angle plate with interlocking screws (TomoFix™, Synthes, Bettlach, Switzerland or Synthes GmbH, Solothurn, Switzerland) and an allogenic bone graft (Junyoung Medical, Seoul, South Korea) to minimize postoperative loss of correction. The amount of correction and the size of the posterior opening gap at the osteotomy site were also determined by aiming for a mechanical femorotibial axis with a valgus overcorrection of 4-5°, which was nearly equivalent to the MA passing through 62 % of the tibial plateau. This estimated opening gap was confirmed by matching the calculated opening gaps using the Miniaci method. Before plate fixation, the valgus correction was confirmed by the navigation system and slight valgus alignment. When deciding the amount of alignment correction based on inspection of the navigation screen monitor, manual compression was performed to minimize any error in the correction arising from the fact that alignment correction was performed under non-weight-bearing conditions. To avoid any increase in tibial posterior slope, we attempted to make the anterior gap of the osteotomy site behind the tibial tuberosity approximately 1/2 to 2/3 of the posterior opening gap at the posteromedial corner of the proximal tibia. In addition, posterior slope was checked by fluoroscopy after valgus correction and just before final plate fixation.

### Radiographic measurements

Preoperative radiographic evaluation consisted of an anteroposterior (AP) lower limb view on standing. These results were used to calculate the MA, defined as the angle subtended by a line drawn from the center of the femoral head to the center of the knee and a line drawn from the center of the knee to the center of the talus. The WBL ratio was determined using a line from the center of the femoral head to the center of the superior articular surface of the talus. The numerator was the tibial intersection of the WBL (with the medial tibial edge at 0 % and the lateral tibial edge at 100 %), and the denominator was the width of the tibia. The angle of the posterior tibial slope on knee lateral radiographs was determined using proximal tibial anatomical axis (PTAA) method [16]. The diaphyseal axis of the tibia was drawn between two points equidistant between the anterior and posterior borders of the tibia, one just below the tibial tubercle and the other 10 cm below this. The articular surface line was drawn from the most superior points at the anterior and posterior edges of the medial tibial plateau. The angle subtended by these two lines was defined as the posterior tibial slope. Standing AP lower limb and knee lateral radiographs were obtained 12 weeks after surgery, when the patient was able to stand without support and could bear full weight on the operated side. Corrections in the coronal plane were calculated as the difference between preoperative and postoperative MA and WBL ratio. Differences between preoperative and postoperative posterior tibial slope were also calculated in the sagittal plane.

### 3 dimensional CT measurement of the anterior and posterior gaps of the osteotomy site

**Fig. 1:**
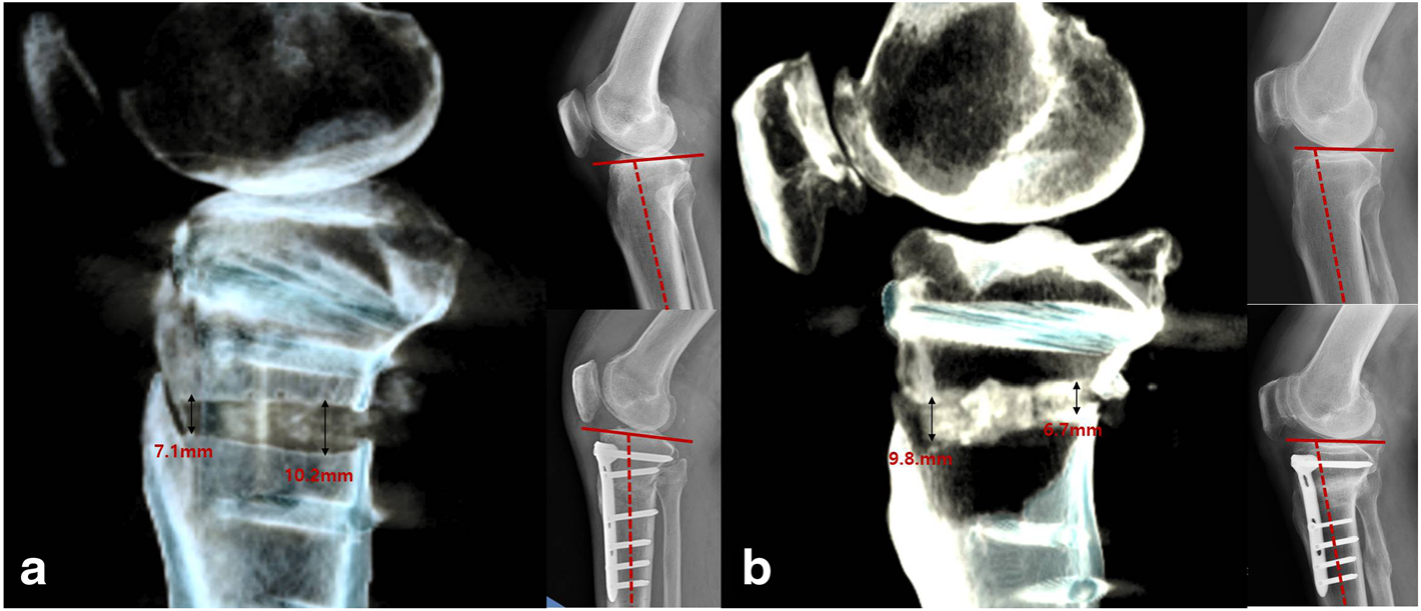
Measurements of anterior and posterior opening gap heights on 3D CT. The opening gap at osteotomy site of the larger posterior gap group, with a significantly larger posterior than anterior gap on postoperative 3D CT, was created as preoperatively planned (**a**). Patients with larger anterior gaps preoperatively had larger anterior than posterior gaps on 3D CT, in opposition to preoperative planning (**b**). Nevertheless, no definite increases in posterior tibial slope were found in both sets of patients

Postoperative multislice CT scanning (MDCT; Brilliance 64, Phillips, Cleveland, OH, USA) was performed using 5 mm coronal, 5 mm sagittal, and 2 mm axial slices of the knee, proximal femur, and distal tibia. Data from the PACS software were imported into Rapidia imaging software (Version 2.8), which provided the operating windows of three multiplanar reformation (MPR) viewers, in the coronal, sagittal, and axial planes. MPR views of the osteotomy surface consisted of the regions 5 cm below the medial joint line to the fibular head in the coronal plane, parallel to the medial tibial plateau in the sagittal plane, and perpendicular to the epicondylar axis in the axial plane. Finally, the osteotomy surface of a coronal section was selected as the reference plane for anterior and posterior slopes on sagittal MPR images. The points of measurement on 3D CT were the posteromedial corner of the osteotomy opening gap for the posterior gap and the anterior 1/3 coinciding with the medial side to the tibial tuberosity for the anterior gap. If, as planned, the posterior gap was larger than the anterior gap at the osteotomy site, the knee was classified as being in the larger posterior gap group. If, however, the anterior gap was larger than the posterior gap, the knee was classified as being in the larger anterior gap group (Fig. 1a, b). All measurements on radiography and 3D CT were performed using a picture archiving and communication system (PACS, PI View STAR, version 5025; Infinitt, Seoul, Korea). All radiographic parameters and anterior and posterior gap heights on 3D CT were independently measured by 2 orthopedic surgeons with significant experience in HTO, and were again measured by both 2 weeks later, with the two measures averaged for analysis.

### Statistical analysis

The primary outcome measure in the present study was the difference between preoperatively planned and postoperative measurements of anterior and posterior gaps on 3D CT. An a priori power analysis was performed to determine a sample size, using the two-sided hypothesis test at an alpha level of 0.05 and a power of 0.8. The results of our pilot study indicated that 38 and 35 knees were required to detect significant differences in anterior and posterior gaps, respectively, for the two measurement methods. We ultimately analyzed 41 knees, indicating adequate power (0.831 for anterior gap and 0.879 for posterior gap to detect significant differences between preoperatively planned and postoperative measurements on 3D CT).

The correlation between preoperatively planned and real osteotomy gaps was calculated by determining the intraclass correlation coefficients (ICCs) between preoperative and postoperative anterior and posterior opening gap heights. ICCs above 0.75 represented good reliability/accuracy, values between 0.4 and 0.75 represented fair reliability, and those below 0.4 represented poor reliability [17]. The inter- and intraobserver differences in radiographic data and opening gaps on 3D CT were also assessed by calculating ICCs. Agreement was assessed by Bland-Altman plots, which provide mean difference and limits of agreement (mean difference ± 1.96 standard deviations). In general, better agreement is indicated by a mean difference closer to 0 and a smaller standard deviation. The significance of systematic differences was assessed using paired t-tests.

Mean between group differences in demographic parameters, gap height measurements on 3D CT, and slope on radiography were compared using Student’s t-test or Mann–Whitney U tests, as appropriate. All statistical analyses were performed using IBM SPSS Statistics version 20 software (IBM Corporation, USA). A *p* value < 0.05 was considered statistically significant.

## Results

### Limb alignment, posterior slope on radiography, and opening gap on 3D CT

The interobserver and intraobserver reliabilities of MA and WBL ratio measurements on full-length standing radiographs and opening gap height measurements on 3D CT ranged from 0.745to 0.817 and from 0.751 to 0.835, respectively, indicating good reliability. The mean preoperative MA was varus 7.2° ± 3.9° (range, 2.0 to 15.5°) and valgus 3.6° ± 2.5° (range, valgus 8.6° to varus 3.2°). The mean MA correction was 10.7° ± 3.9° (range, 2.6 to 18.1°). The mean WBL ratio was 17.7 % ± 14.8 % (range, 2.5 to 47.5 %) preoperatively and 64.2 % ± 12.6 % (range, 36.5 to 86.3 %) postoperatively. The mean change in WBL ratio following HTO was 46.4 % ± 15.9 % (range, 10.2 to 75.4 %). Mean preoperative [10.8° ± 2.8°, (range, 4.5 to 21.5°)] and postoperative [10.5° ± 3.3°, (range, 5.3 to 20.1°)] posterior slopes of the tibia were identical (p = 0.556).

Across all patients, the mean posterior opening gap was significantly greater on preoperative planning than on postoperative 3D CT (12.2 ± 4.0 mm vs. 11.2 ± 3.5 mm, *p* = 0.033). In contrast, the mean anterior opening gap was significantly smaller preoperatively planned than postoperatively measured (8.53 ± 2.8 mm vs. 10.8 ± 3.5 mm, *p* < 0.001, Table 1).

**Table 1:** Mean difference (systematic bias) between real measurements on computed tomography and calculated data on opening gaps

	CT		Calculation		(C)-(D)	
	Mean (C)	SD	Mean (D)	SD		*P*-value
Anterior gap	10.84	3.48	8.53	2.77	2.30	<0.001
Posterior gap	11.18	3.50	12.19	3.97	−1.02	0.033
Δ gap (ant-post gap)	0.34	2.10	3.66	1.19	−3.32	<0.001

### Sagittal tibial slope in patients with larger anterior and posterior gaps

Of the 41 knees, 24 (59 %) had larger posterior than anterior gaps on postoperative 3D CT, whereas the other 17 (41 %) had larger anterior than posterior gaps. The demographic characteristics of the two groups were similar (Table 2). Interestingly, preoperative slope, postoperative slope, and change in slope were similar in the two groups (Table 3).

**Table 2:** Demographic characteristics of all subjects, classified as those with larger anterior than posterior gaps

	Overall	Larger anterior gap group	Larger posterior gap group	*P*-value
Sample size (number)	41	18	23	
Gender (male/female)	10/31	3/15	7/16	0.467
Age (years)	56.8 ± 7.6	57.6 ± 7.1	56.2 ± 8.0	0.538
Height (cm)	158.6 ± 8.0	159.3 ± 7.2	158.1 ± 8.6	0.648
Weight (kg)	65.9 ± 9.9	66.6 ± 10.3	65.4 ± 9.7	0.703
Body mass index (kg/m^2^)	26.2 ± 3.1	26.2 ± 3.5	26.1 ± 2.9	0.122

**Table 3:** Gap measurement parameters in larger anterior and posterior gap groups

	Larger anterior gap group (*n* = 17)	Larger posterior gap group (*n* = 24)	*P*-Value
Anterior gap (mm)	12.40 ± 3.38	9.61 ± 3.10	0.009
Posterior gap (mm)	10.77 ± 3.23	11.49 ± 3.73	0.518
Gap difference (posterior-anterior gap, mm)	−1.62 ± 1.21	1.88 ± 1.11	<0.001
Preoperative tibial slope (°)	10.13 ± 2.56	11.32 ± 2.98	0.188
Postoperative tibial slope (°)	9.88 ± 2.80	11.03 ± 3.66	0.278
Slope change (postoperative-Preoperative tibial slope, °)	−0.25 ± 3.25	−0.29 ± 2.71	0.969

### Correlation between preoperatively planned and postoperatively measured opening gap data

**Fig. 2:**
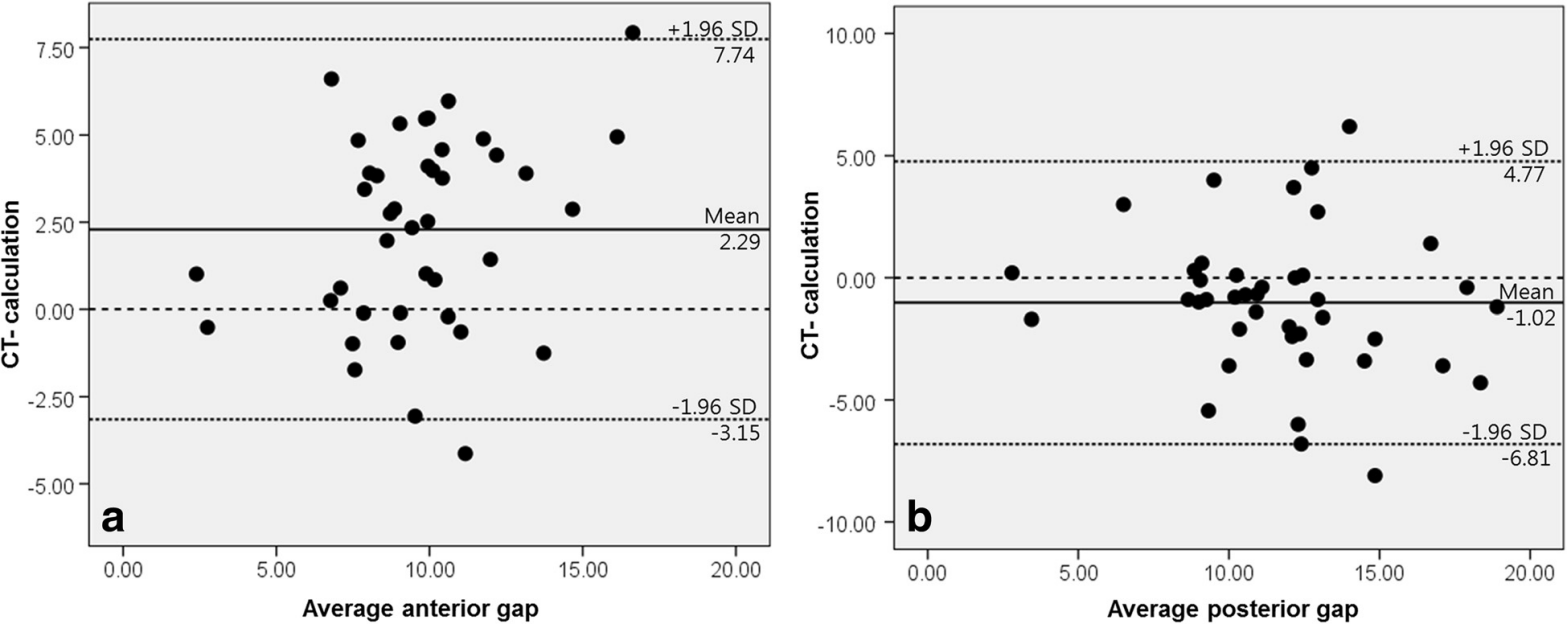
Bland-Altman plots of differences between preoperative planned and postoperatively measured anterior (**a**) and posterior (**b**) opening gap heights on 3D CT. Solid line, mean difference; short broken line, mean difference ± 1.96 standard deviations (SDs); long broken line, zero value of x-axis

The ICCs between preoperative planned and postoperatively measured opening gap heights are summarized in Table 4. Although correlations were good for posterior gap measurements, those for anterior gap measurements were only fair. Bland-Altman plots comparing preoperatively planned and postoperatively measured opening gaps showed poor agreement for both anterior and posterior gaps (Fig. 2a, b). The mean systematic differences (bias) were 2.3 mm (*p* < 0.001) for anterior and -1.0 mm (*p* = 0.033) for posterior gaps. The limits of agreement were too wide for both anterior (–3.2 ~ 7.7 mm) and posterior (–6.8 ~ 4.8 mm) gaps.

**Table 4:** Intra-class correlation coefficients (ICCs) between real measurements on computed tomography and calculated opening gaps

	ICC	95 % CI for ICC		*P*-value
		LB	UB	
Anterior gap	0.611	0.104	0.846	<0.001
Posterior gap	0.815	0.622	0.895	<0.001
Δ gap (ant-post gap)	0.181	−0.535	0.563	0.265

*LB* lower border, *UB* upper border

## Discussion

This study evaluated the agreement between preoperative planned and postoperatively measured anterior and posterior opening gaps in medial opening HTO. Preoperative planning was designed so that the posterior gap would be larger than the anterior gap, minimizing changes in posterior tibial slope after surgery; however, these gaps did not correspond with the actual postoperative opening gaps measured on 3D CT. Posterior tibial slope was similar in groups of patients with larger posterior and anterior gaps.

Tibial slope has been reported to increase inadvertently after open wedge HTO due to distinguishing anatomical features of the cross-sectional shape of the proximal tibia [11, 13]. Because the anteromedial cortex of the proximal tibia forms a 45° angle with the posterior cortex, whereas the lateral cortex is perpendicular to the posterior plane of the tibia, an opening wedge with equal anterior and posteromedial gaps would tend to increase the sagittal tibial slope, suggesting that, to avoid unintentional posterior tibial slope, the anterior gap should be one-half to two-thirds the size of the posteromedial gap [13, 14]. A method of avoiding unintentional increases in posterior slope in 40 subjects who underwent navigation-assisted open wedge HTO included making the anterior opening gap around the tibial tuberosity about 67 % the size of the posterior opening gap [14]. This surgical opening gap planning achieved satisfactory correction in the coronal plane without inadvertently increasing the posterior tibial slope in the sagittal plane. Virtual open wedge HTO in 35 computer-aided models of the proximal tibia resulted in an anterior osteotomy gap at the tibial tubercle being about one half the size of the posteromedial gap to maintain a normal sagittal tibial slope [13]. Evaluation of the change in posterior tibial slope after open wedge HTO using autologous cortical iliac bone grafts included keeping the anterior gap of the osteotomy site at approximately one-half or two-thirds of the posterior gap, finding that posterior tibial slope did not change significantly [18]. These three studies, however, assessed tibial slope change after open wedge HTO radiographically, but did not show directly that the real opening gap after surgery had been created as planned, indicating that the anterior opening gap around the tibial tubercle was about one-half to two-thirds that of the posterior opening gap at the osteotomy site.

Our measurements of the anterior and posterior opening gaps at the osteotomy site using in vivo 3D CT showed that, of the 41 subjects, 17 (41 %) had a larger anterior than posterior opening gap at the osteotomy site, indicating that the actual opening gap was not always created as calculated preoperatively. In addition, patients with larger anterior than posterior gap heights did not show an increase in posterior slope, a finding in contrast to the results of previous studies.

Although the mechanism responsible for this lack of association between larger anterior than posterior opening gap height and increased posterior tibial slope is unclear, it may be associated with several factors that can lead to tibial slope change after HTO. Incomplete osteotomy of the posterior cortex and partial release of posterior soft tissue may increase the posterior tibial slope [19]. Inadequate posterior corticotomy and soft tissue release may be due to surgeons’ concerns for posterior neurovascular injury during open wedge HTO. Moreover, it is difficult to determine the lateral hinge location of open wedge HTO. The direction of the cutting blade is dependent on the sagittal alignment of the cutting jig [20], which is difficult to control during surgery and affects the posterior slope. Change in posterior slope was found to be larger in the posterolateral than in the lateral cortical hinge, because of the tendency to insert the pin in the posterolateral rather than the lateral direction, a tendency due to the oblique configuration of the anteromedial proximal tibial cortex [21]. The location of plate fixation can also affect the posterior tibial slope. The anteromedial position of the plate increased the tibial slope by 4.3° compared with only 1.0° for the posteromedial plate position [22]. A more anterior plate location is associated with a more posterolateral hinge location, tending to increase the posterior slope. Anatomical variations of the proximal tibia shape may also influence the posterior slope. The preoperative posterior slope may be preserved after HTO by making the required anterior osteotomy gap at the tibial tubercle approximately one-half to two-thirds the posteromedial gap. This equation is predicated on the assumption that the obliquity of the anteromedial to the posterior cortex is 45° on a cross-section of the proximal tibia. Thus, this equation is not applicable if the oblique angle of the anteromedial cortex in the proximal tibia is not 45°, reducing the accuracy of preoperative planning to maintain the posterior slope. Soft tissue laxity around the knee joint associated with dynamic gait alignment may also affect the posterior tibial slope, given that the soft tissue laxity quantified by the joint line convergence angle was correlated with the amount of limb alignment correction from before to after open wedge HTO [23]. Finally, even if the anterior gap is larger than the posterior gap, the difference between them may be too small to increase the posterior tibial slope.

This study had several limitations. Clinical outcomes were not evaluated, but that was not the primary aim of this study. Another limitation was the lack of use of preoperative 3D CT as a baseline for the posterior tibial slope in the sagittal plane. Exposure to radiation during CT scans could result in various detrimental effects [24], although we had informed the included subjects of the deleterious effects of exposure to radiation. To reduce radiation exposure, preoperative CT was not performed. Finally, 3D CT images were obtained with subjects in the nonweight bearing supine position. Thus, we could not entirely exclude the possibility of secondary slope change after weight bearing due to severe osteoporosis or inadequate fixation.

## Conclusions

Preoperative planning to prevent changes in posterior tibial slope during medial opening wedge HTO was designed to make the anterior gap at the osteotomy site about 1/2 to 2/3 the posterior opening gap, but did not always correlate with actual postoperative opening gaps, as assessed by in vivo 3D CT. Therefore, surgeons should take care to keep the anterior gap at 1/2 to 2/3 of the posterior gap throughout open wedge HTO.

## Abbreviations

CT: computed tomography
HTO: high tibial osteotomy
MA: mechanical axis
PTAA: proximal tibial anatomical axis
WBL: weight-bearing line
